# IFIT2 Depletion Promotes Cancer Stem Cell-like Phenotypes in Oral Cancer

**DOI:** 10.3390/biomedicines11030896

**Published:** 2023-03-14

**Authors:** Kuo-Chu Lai, Prabha Regmi, Chung-Ji Liu, Jeng-Fan Lo, Te-Chang Lee

**Affiliations:** 1 Department of Physiology and Pharmacology, College of Medicine, Chang Gung University, Taoyuan City 33302, Taiwan; 2 Graduate Institute of Biomedical Sciences, College of Medicine, Chang Gung University, Taoyuan City 33302, Taiwan; 3 Division of Hematology and Oncology, Department of Internal Medicine, New Taipei Municipal TuCheng Hospital, New Taipei City 23652, Taiwan; 4 Institute of Biomedical Sciences, Academia Sinica, Taipei 11529, Taiwan; 5 Department of Oral and Maxillofacial Surgery, Mackay Memorial Hospital, Taipei 10421, Taiwan; 6 Institute of Oral Biology, National Yang Ming Chiao Tung University, Taipei 11221, Taiwan

**Keywords:** interferon-induced proteins with tetratricopeptide repeats 2, oral cancer, cancer stem cells

## Abstract

(1) Background: Cancer stem cells (CSCs) are a small cell population associated with chemoresistance, metastasis and increased mortality rate in oral cancer. Interferon-induced proteins with tetratricopeptide repeats 2 (IFIT2) depletion results in epithelial to mesenchymal transition, invasion, metastasis, and chemoresistance in oral cancer. To date, no study has demonstrated the effect of IFIT2 depletion on the CSC-like phenotype in oral cancer cells. (2) Methods: Q-PCR, sphere formation, Hoechst 33,342 dye exclusion, immunofluorescence staining, and flow cytometry assays were performed to evaluate the expression of the CSC markers in IFIT2-depleted cells. A tumorigenicity assay was adopted to assess the tumor formation ability. Immunohistochemical staining was used to examine the protein levels of IFIT2 and CD24 in oral cancer patients. (3) Results: The cultured IFIT2 knockdown cells exhibited an overexpression of ABCG2 and CD44 and a downregulation of CD24 and gave rise to CSC-like phenotypes. Clinically, there was a positive correlation between IFIT2 and CD24 in the patients. IFIT2^high^/CD24^high^/CD44^low^ expression profiles predicted a better prognosis in HNC, including oral cancer. The TNF-α blockade abolished the IFIT2 depletion-induced sphere formation, indicating that TNF-α may be involved in the CSC-like phenotypes in oral cancer. (4) Conclusions: The present study demonstrates that IFIT2 depletion promotes CSC-like phenotypes in oral cancer.

## 1. Introduction

Oral cancer is the sixth most prevalent malignancy worldwide, and its incidence varies depending on the geographic region [1,2]. Oral squamous cell carcinoma (OSCC) is highly malignant and is associated with cervical nodal and distant metastasis in 25–65% of patients [3]. Despite the advancements in diagnosis and therapeutics, the overall five year survival rate of oral cancer patients has remained at 50–60% for decades [4,5]. Treatment failure and eventually mortality in oral cancer patients is in general due to the recurrence, metastasis, and chemo/radioresistance that are associated with a small number of cancer stem cells (CSCs) or tumor-initiating cells [6].

CSCs are capable of sustaining tumor growth in situ and possess self-renewal and differentiation abilities and seed tumors in xenograft models [7]. CSCs have been reported in both primary oral cancer and cell lines and are resistant to various chemotherapeutic agents, such as cisplatin, docetaxel, etoposide, gemcitabine, paclitaxel, carboplatin, and 5-fluorouracil (5-FU) [6,8]. CSCs are capable of cellular plasticity, where they can switch between the epithelial to mesenchymal transition (EMT) differentiation program and the reverse program [9]. Consequently, various studies have reported the role of phenotypic plasticity in cancer initiation, progression, metastasis, and resistance to therapy in various primary and metastatic neoplasms, such as lung, prostate, pancreatic, and head and neck cancers (HNCs) [10,11,12,13,14,15,16]. Numerous studies also suggest that cells undergoing EMT have the potential to acquire the CSC characteristics of self-renewal and to reprogram the gene expression associated with stemness [9,17,18]. However, studies on the pathogenesis and molecular mechanism of CSCs in oral cancer are limited and require further investigation [19].

There are four members in the interferon-induced proteins with the tetratricopeptide repeat (IFIT) family (IFIT1, IFIT2, IFIT3 and IFIT5) that display a broad spectrum of antiviral functions and are involved in various biological processes, such as proliferation, migration, translational initiation, and RNA signaling [20,21]. Emerging studies have shown the importance of IFITs in the signaling pathways involved in cancer progression, metastasis, and drug resistance [22]. Intriguingly, although IFITs share conserved structural motifs, their isoforms display differential expression and nonredundant functions [23,24]. Elevated levels of IFIT1, IFIT3 and IFIT5 have been shown to play significant roles in cancer progression, while the decreased expression of IFIT2 has been reported to enhance invasion, tumor progression, and drug resistance in various cancer types [25,26,27,28,29,30,31,32]. Furthermore, increased IFIT1 and IFIT3 expression but decreased IFIT2 expression is correlated with poor survival in OSCC patients [25,33]. On the contrary, increased IFIT2 expression inhibits cell proliferation and triggers apoptosis in various cancer types [34,35,36,37]. The IFIT family may play a critical role in tumor progression in various types of cancer.

We have previously shown that IFIT2 knockdown leads to the activation of atypical protein kinase C (PKC) signaling, accompanied by EMT and an increase in migration, invasion, distant metastasis, chemoresistance, and TNF-α expression, and eventually results in angiogenesis and poor survival in oral cancer [31,32,33,38]. The accumulating evidence shows that cells undergoing EMT have CSC characteristics and that EMT is an important bridge between metastasis, drug resistance, and CSCs [9,39,40]. Whether IFIT2-depleted oral cancer cells exhibit a CSC-like phenotype is still unknown. In the present study, we aimed to assess the CSC properties in IFIT2 knockdown and control cells. Examining the relationship between IFIT2 depletion and CSCs may be helpful for proposing an approach to block cancer progression, recurrence, and metastasis, to overcome drug resistance, and to stratify patients based on optimal treatment regimens.

## 2. Materials and Methods

### 2.1. Cell Culture and Reagents

Sh-control and stable IFIT2-depleted (sh-IFIT2-1 and sh-IFIT2-2) cells were generated in the human oral cancer cell line CAL27 as previously described [31]. The cells were screened for mycoplasma contamination following the manufacturer’s instructions (abm Mycoplasma PCR detection kit; Cat no. G238 Applied Biological Materials Inc. Richmond BC, Canada) and cultured as previously described [32]. We also tried to knockdown IFIT2 in other cell lines. However, based on the IFIT2 expression levels, CAL27 cells were apparently the most convenient cells for this study.

### 2.2. Western Blot Analysis

Western blot analysis was performed to determine the expression level of proteins as previously described [32]. The primary antibodies, anti-IFIT2 (sc-390724), anti-OCT3/4 (sc-5279), anti-NANOG (sc-293121), and anti-ABCG2 (sc-58222), were obtained from Santa Cruz Biotechnology (Santa Cruz, CA, USA); anti-TNFα (#6945) from Cell Signaling Technology (Danvers, MA, USA); anti-GAPDH (#60004-1-Ig) and anti-β-actin (66009-1-Ig) from Proteintech (Chicago, IL, USA). Anti-rabbit-HRP (#ab97051) and anti-mouse-HRP (#205719) were purchased from Abcam (Cambridge, UK).

### 2.3. Anchorage-Independent Growth Assay

The soft agar assay is a common method to examine anchorage-independent cell growth to validate transformed cells [41]. To evaluate the clonogenicity of IFIT2 knockdown cells on soft agar, the bottom layer of the 60 mm dish was coated with 0.7% agar prepared in 2× DMEM supplemented with 4% peptone and FBS. After gel solidification, 3 mL of 0.3% top agar containing 1000 cells per well was added and incubated at 37 °C for 6 weeks. During the course of incubation, 500 µL of fresh medium was added every 3–4 days. The cells were stained with 750 µL of 1 mg/mL iodonitrotetrazolium (INT) for 2–3 days. Images were taken under the microscope at 10× magnification, and the colonies were counted.

### 2.4. Sphere Formation Assay

To evaluate the sphere growth and formation, the cells were plated at a density of 1000 cells per well in ultralow attachment 6-well plates (Corning Inc., Corning, NY, USA) and cultured in serum-free DMEM-F12 medium supplemented with 1% B27 supplement, 20 ng/mL epidermal growth factor (EGF) and 20 ng/mL basic fibroblast growth factor (bFGF) for 2 weeks. EGF and bFGF were purchased from PeproTech, Rocky Hill, NJ, USA. The sphere formation was assessed by counting the spheres (>50 µm) under a microscope.

### 2.5. Side Population (SP) Assay

The SP was quantified by a Hoechst dye exclusion assay [42]. The OSCC cells (10^6^/mL) were incubated with Hoechst 33,342 dye (5 µg/mL) in the presence or absence of verapamil (50 µM) and incubated at 37 °C in a water bath for 90 min with intermittent shaking. Then, the cells were washed with ice-cold phosphate-buffered saline (PBS) with 2% FBS and resuspended in 2 µg/mL propidium iodide prepared in ice-cold PBS containing 2% FBS. The SP population was quantified using fluorescence-activated cell sorting (FACS) in the IFIT2 knockdown cells and compared to vector control cells, as well as verapamil-treated and nontreated cells. The Hoechst 33,342 dye was excited, and its fluorescence at dual wavelengths was analyzed (blue, 402–446 nm; red, 650–670 nm). Hoechst dye was purchased from BD Bioscience (San Jose, CA, USA).

### 2.6. Quantitative Real-Time PCR (QPCR)

Q-PCR was performed as previously described [32]. The primer sequences are listed in Supplementary Table S1.

### 2.7. Flow Cytometry Analysis

The growing cells cultured in 100 mm dishes were washed with 1× PBS, trypsinized, centrifuged, and resuspended in 2% FBS in Hank’s balanced salt solution (HBSS). The viable cell number was counted using an automatic cell counter (trypan blue exclusion), and the cell density was adjusted to 1 × 10^6^ cells/100 μL. For CD24 and CD44 staining, 20 μL of antibodies (anti-CD24, anti-CD44 and PE-mouse IgG isotype control and APC-mouse IgG isotype control) were added to 100 μL of cell suspension and incubated at 4 °C for 30 min per the manufacturer’s protocol. However, for ABCG2 staining, 5 μL of APC Mouse Anti-Human CD338 and APC-mouse-IgG isotype control were added to 100 μL cell suspension and incubated at 4 °C for 30 min, according to the manufacturer’s instructions. After incubation, the cells were washed three times with HBSS and centrifuged at 2000 rpm for 3 min. Then, the cells were resuspended in 300 μL 1× PBS containing 1 μg/mL DAPI and were passed through tube filters to avoid cell aggregation. Finally, the cells were analyzed using flow cytometry. PE mouse anti-human CD24, APC mouse anti-human CD44, PE mouse IgG2α, κ isotype control (555574), APC mouse IgG2α, and κ isotype control antibodies were purchased from BD Bioscience (San Jose, CA, USA), and anti-mouse IgG_1_ isotype control (MAB002) was purchased from R & D Systems, Inc. (Minneapolis, MN, USA).

### 2.8. Immunofluorescence and Confocal Microscopy

The intracellular location of ABCG2, CD24 and CD44 was examined by immunofluorescence staining. The cultured sh-control and stable IFIT2 knockdown cells were fixed with 100% ice-cold methanol and permeabilized with 0.2% Triton X-100 and incubated with the ABCG2, CD24, and CD44 antibodies. Anti-mouse IgG secondary antibody Alexa Fluor 555 (Molecular Probes, Eugene, OR, USA) was then added and incubated for 1 h. The cell nuclei were stained with 4′,6-diamidino-2-phenylindole (DAPI). After mounting the slides with 50% glycerol in PBS, images were acquired under a laser scanning confocal microscope (Carl Zeiss MicroImaging Inc., Thornwood, NY, USA) and Axio Vision software.

### 2.9. Tumorigenicity Assay

In vivo tumorigenesis was performed according to the guidelines of experimental animals and approved by the Institutional Animal Care and Utilization Committee (IACUC) of Academia Sinica. The mice were housed in a specific pathogen-free environment under 12 h light-dark cycles in an animal core facility at the Institute of Biomedical Sciences, Academia Sinica, Taipei, Taiwan. To determine the tumorigenicity, the sh-control and stable IFIT2 -depleted cells were subcutaneously implanted into the dorsal flank region of 5-week-old male BALB/c nude mice. Different numbers of cells (10^5^, 5 × 10^4^, 10^4^, 5 × 10^3^, 10^3^, 500, 100, 50, 25 and 10 cells) were mixed with Matrigel (1:1 volume) to generate a 100 µL cell-Matrigel mixture. Matrigel was purchased from Corning (Two Oak Park, Bedford, MA, USA). The mice were housed under observation until the tumor was seen. Tumor volume was measured using the formula: Volume = 1/2 (length × width^2^). Tumor formation was confirmed using hematoxylin and eosin (H & E) stained sections.

### 2.10. Immunohistochemical Staining

Oral cancer patient tissue samples were obtained and analyzed, abiding the rules and approval of Mackay Memorial Hospital’s institutional review board and the IRB of the Institute of Biomedical Research, Academia Sinica. In brief, the slides with tissue sections were deparaffinized in xylene for 7 min, twice, and then rehydrated in graded ethanol serially from 100% to 70%, followed by rinsing with distilled water. The deparaffinization process was conducted by the Pathology Core of IBMS, Academia Sinica. Subsequently, the slides were immersed in citrate buffer (0.01 M, pH 6.0) and boiled for 50 min in a cooker for antigen retrieval, followed by cooling for 30 min at ambient temperature. Then, the slides were rinsed in distilled water again before immunohistochemistry was performed. The staining procedure was conducted using the Novolink Polymer Detection System (Leica Biosystems, Wetzlar, Germany) following the manufacturer’s instructions. The primary antibody dilution for both the IFIT2 and CD24 was used at a ratio of 1:100. Anti-CD24 antibody was purchased from Santa Cruz Biotechnology (Santa Cruz, CA, USA). Then, the immunostained tissue section slides were scanned using a Panoramic 250 Flash II whole slide scanner at high magnification. The expression levels of IFIT2 and CD24 were analyzed using the 3DHISTECH Panoramic viewer software (3DHISTECH Ltd., Budapest, Hungary).

### 2.11. Enzyme-Linked Immunoassay (ELISA)

A total of 3 × 10^5^ and 5 × 10^5^ cells were seeded in 6-well plates and cultured in serum-free medium for 48–72 h. The collected conditioned medium was used to perform an ELISA as per the manufacturer’s instructions. The absorbance of the standard was compared to that of the test sample to quantify the TNF-α concentration. The Human TNF-α Quantikine ELISA kit (DTA00C) was purchased from R & D Systems, Inc. (Minneapolis, MN, USA).

### 2.12. Statistical Analysis

The data analysis was performed using the GraphPad software (version 6.0). To identify significant differences between the two different groups, Student’s *t*-test was used. The body weight and tumor volume between the animal groups were compared using a one-way ANOVA. *p* values < 0.05 were considered to indicate statistical significance. The correlation and significance of differences for immunohistochemistry were determined by the Pearson correlation coefficient test.

## 3. Results

### 3.1. IFIT2 Knockdown Cells Exhibit CSC-like Properties

**Figure 1:**
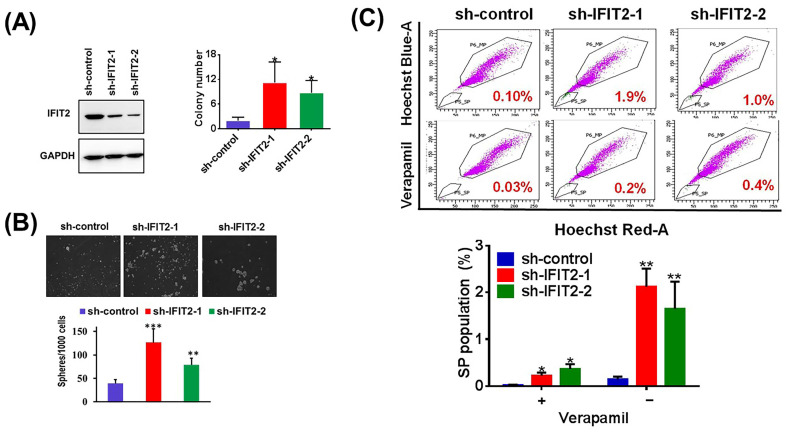
In vitro soft agar colony and spheroid formation in stable IFIT2-depleted cells. (**A**) The IFIT2 protein levels in sh-control and stable IFIT2 knockdown cells were determined by Western blotting. GAPDH expression was used as a loading control (left panel). The results of the anchorage-independent colony formation assay were shown in right panel; (**B**) Representative image of spheroid formation and quantitated data of spheroid numbers counted in three independent experiments in sh-control and stable IFIT2 knockdown cells; (**C**) Representative flow cytometry of SP assay with or without verapamil treatment (upper). The quantitative analysis of the SP population was average of three independent experiments (bottom). The symbols *, ** and *** indicate statistically significant differences compared to sh-control at *p* < 0.05, *p* < 0.01 and *p* < 0.001, respectively.

Self-renewal and differentiation are the main features of CSCs and are commonly assessed by analyzing the anchorage-independent growth and spheroid abilities. We first confirmed the low expression of IFIT2 in the IFIT2-depleted cells using Western blotting (Figure 1A). Similarly, a clonogenicity assay showed that the IFIT2 knockdown increased the anchorage-independent growth compared to the sh-control cells (Figure 1A). Moreover, we analyzed the spheroid formation ability of the IFIT2 knockdown cells using a sphere formation assay. The Sh-IFIT2-1 and sh-IFIT2-2 cells showed a significantly higher number of spheroids than the sh-control cells, indicating an enhanced self-renewal capacity in the IFIT2 knockdown cells (Figure 1B). CSCs have a higher drug efflux capacity, mainly due to their high expression levels and the activity of drug transporters such as ABCB1 and ABCG2 [43,44]. Using the Hoechst 33,342 dye exclusion assay, we observed an enriched SP in the stable IFIT2-depleted cells compared to the sh-control cells. Approximately 2% of the stained population was designated as the SP group in the IFIT2 knockdown cells (Figure 1C).

### 3.2. Characterization of CSC Markers in IFIT2 Knockdown Cells

**Figure 2:**
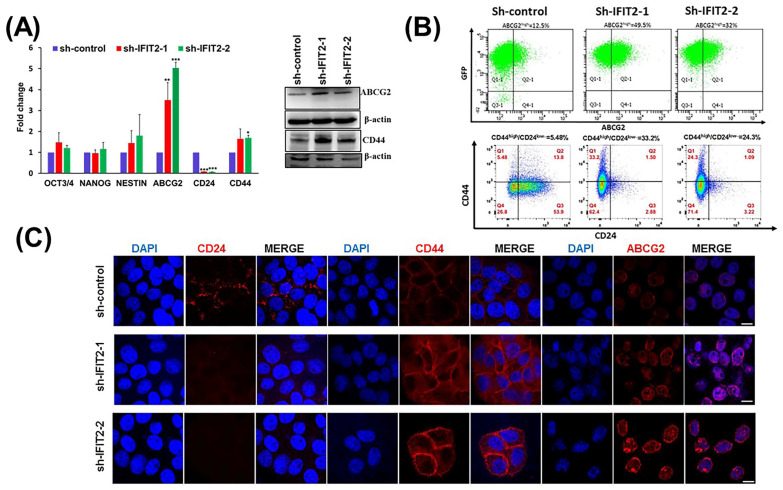
Expression of CSC markers in IFIT2 knockdown cells. (**A**) The mRNA expression levels of CSC markers in sh-control and stable IFIT2 knockdown cells were determined by Q-PCR, while the protein levels were analyzed by Western blotting. GAPDH was used as an internal control in Q-PCR and β-actin was used as an internal control in Western blotting; (**B**) Representative flow cytometry analysis of ABCG2, CD44, and CD24 in sh-control and IFIT2-depleted cells. Experiments were performed four times; (**C**) Representative image of immunofluorescence of ABCG2, CD44 and CD24 staining in sh-control and stable IFIT2 knockdown cells. Rhodamine-conjugated secondary antibody for ABCG2, CD24 and CD44 (red) were applied, and nuclei were counterstained with DAPI (blue). Scale is 10 µm. The symbols *, ** and *** indicate statistically significant differences compared to sh-control at *p* < 0.05, *p* < 0.01 and *p* < 0.001, respectively.

Our unpublished cDNA microarray data showed that several probes targeting CD44 or ABCG2 were significantly enhanced, whereas CD24 was decreased, in the IFIT2 knockdown cells compared with the control cells. Other CSC markers, such as NANOG, SOX2, and KLF4, were not different among them (Supplementary Figure S1). Accordingly, Q-PCR and Western blot analysis were conducted to characterize the CSC features in the IFIT2 knockdown cell. We confirmed that the mRNA levels of ABCG2 and CD44 were significantly increased in the IFIT2 knockdown cells compared with the sh-control cells, whereas the mRNA of CD24 were significantly lower in the IFIT2-depleted cells. The protein levels of CD44 and ABCG2 were consistent with the relative mRNA expression (Figure 2A). The mRNA and protein levels of Nanog and Oct-4 were not significantly different between these cells (Figure 2A and Supplementary Figure S2). Furthermore, we evaluated the population of ABCG2, CD44, and CD24 expression in these cells using flow cytometry. The ABCG2-positive populations accounted for 49.5% and 32% of the sh-IFIT2-1 and sh-IFIT2-2 cells, respectively, which was an increase compared to the proportion in the sh-control cells (12.5%). Similarly, double staining for CD44 and CD24 was performed to analyze the CD44^+^/CD24^−^, CD44^+^/CD24^+^, CD44^−^/CD24^−^, and CD44^−^/CD24^+^ populations. The proportions of CD44^high^/CD24^low^ cells was 5.48% in the sh-control cells; however, CD44^high^/CD24^low^ cells were 33.2% and 24.3%, respectively, observed in the sh-IFIT2-1 and sh-IFIT2-2 cells (Figure 2B). These results depict the enrichment of CD44 high and CD24 low populations in the IFIT2-depleted cells, showing the potential role of IFIT2 knockdown in harboring CSC-like properties. In addition, the intracellular localization of CD44, CD24 and ABCG2 was visualized using immunofluorescence staining. As expected, ABCG2 and CD44 were highly expressed in the stable IFIT2-depleted cells compared to the sh-control cells, whereas CD24 expression showed minimal staining in the IFIT2 knockdown cells (Figure 2C). These results validate that IFIT2 depletion may enhance the CSC population in oral cancer cells.

### 3.3. Tumorigenicity in IFIT2 Knockdown Cells

**Figure 3:**
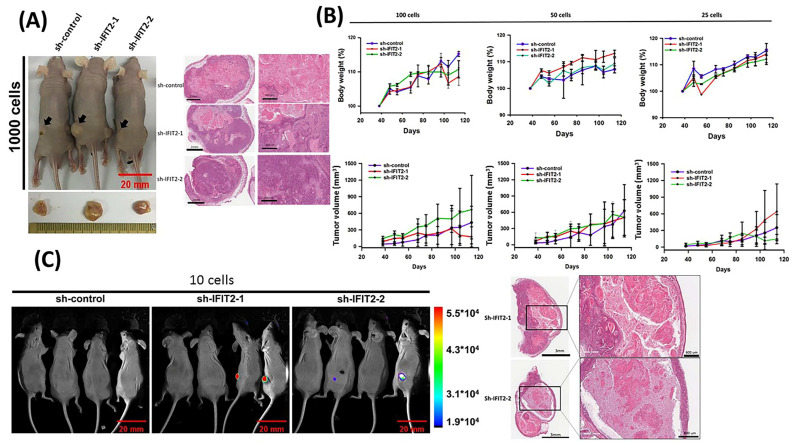
In vivo tumorigenicity of stable IFIT2-depleted cells. (**A**) Representative image of mice and H & E staining of primary xenograft tumors generated by injecting 1000 cells subcutaneously into nude mice; (**B**) Body weight and tumor volumes were monitored throughout the indicated period. There were no statistically significant differences in body weight and tumor volume between groups. (**C**) Images of mice bearing tumors 114 days after implantation of 10 cells. The xenograft tumors were visualized by the IVIS system, and pathology was assessed by H & E staining.

The in vivo tumor-initiating properties of cells are also hallmarks of CSCs; hence, we performed an in vivo tumorigenicity assay by subcutaneously transplanting the stable IFIT2 knockdown and sh-control cells into the flanks of the nude mice. As shown in Table 1 and Figure 3A, these three sublines had a 100% success rate when more than 1000 cells were transplanted subcutaneously into the mice. The body weights and tumor volumes were not significantly different between these sublines (Figure 3B). The success rate decreased when the number of cells was reduced, but there was no apparent difference between the three sublines until the seeding numbers reached at least 25 and 10 cells. In particular, when the cell inoculation number was at least ten cells, there was no tumor growth in the sh-control group, but tumor formation was still observed in the IFIT2 knockdown cells (Figure 3C). The results show enriched tumorigenesis in the IFIT2 knockdown cells.

### 3.4. Clinical Signature of IFIT2 and CSCs in HNC

**Figure 4:**
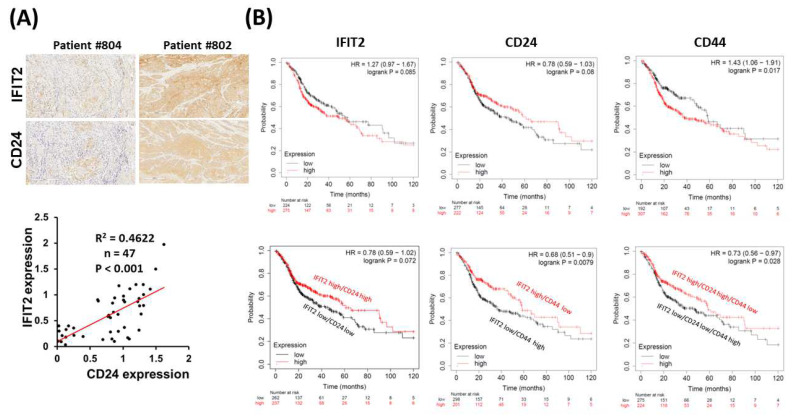
Clinical value of IFIT2 and CSC markers. (**A**) Representative images of low and high immunostaining for IFIT2 and CD24 proteins in OSCC patients (patient IDs: #804 and #802). Correlation between IFIT2 and CD24 expression in OSCC. The *p* value was determined by the Pearson correlation coefficient test; (**B**) Kaplan–Meier plotter analysis of overall survival in 499 HNC patients grouped according to IFIT2, CD44, and CD24 expression. The data were obtained from TCGA.

IFIT2 knockdown strongly promoted the properties of CSCs; however, the clinical correlation of IFIT2 and CSCs is still unknown. With the aid of IHC, the IFIT2 and CD24 protein levels in the 47 primary OSCC tissues were evaluated and showed a positive correlation between IFIT2 and CD24 (*p* < 0.001) (Figure 4A). In addition, we estimated the prognostic effect of IFIT2, CD24, and CD44 in patients with HNC from the TCGA database. Cox regression analysis was performed using the RNA-seq expression of IFIT2, CD24, and CD24 in HNC patients. The correlations between IFIT2, CD24, and CD24 and survival were computed using Cox proportional hazards regression and by plotting Kaplan–Meier survival plots [45]. In the 499 HNC patients, the expressions of IFIT2 or CD24 did not show an association with overall survival; however, CD44 was associated with the overall survival (*p* = 0.017; Figure 4B). The IFIT2^high^/CD24^high^ expression profile was associated with better overall survival in HNC patients (the medium follow-up overall survival was 61.27 months compared to 46.47 months; *p* = 0.072); the IFIT2^high^/CD44^low^ group had a significantly better overall survival rate (the medium follow-up overall survival was 58.73 compared to 36.43 months; *p* < 0.01); and the IFIT2^high^/CD24^high^/CD44^low^ expression also predicted a better survival rate (the medium follow-up overall survival was 58.73 compared to 46.6 months; *p* = 0.028). These results demonstrate that IFIT2 and CSC markers may be prognostic factors in HNC.

### 3.5. Effect of a TNF-α Inhibitor on Spheroid Formation in IFIT2 Knockdown Cells

**Figure 5:**
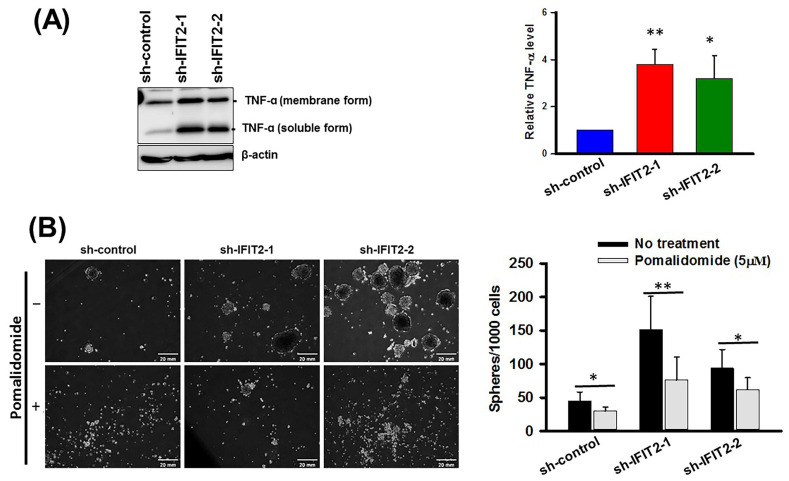
Effect of pomalidomide on spheroid formation in stable IFIT2 knockdown cells. (**A**) The TNF-α protein levels in sh-control and stable IFIT2 knockdown cells were determined by Western blotting (left panel) and ELISA (right panel). β-actin expression was used as an internal control in Western blotting; (**B**) Representative image of spheres with or without pomalidomide (5 µM). The quantitative data were average of seven independent experiments. The symbols * and ** indicate statistically significant differences at *p* < 0.05 and *p* < 0.01, respectively.

Our previous study showed increased TNF-α expression in IFIT2-depleted metastatic and xenograft-derived sublines, and its inhibition resulted in decreased tumor growth, abolished angiogenic activity, and inhibited metastasis [38]. Inflammatory cytokines (interferons, TNF-α, IL-6, and IL-17) and inflammatory cells modulate the gene expression that regulates survival, proliferation, self-renewal, metastasis, and cancer stemness. As expected, increased TNF-α was detected in the IFIT2-depleted cells compared to the sh-control cells (Figure 5A). Furthermore, pomalidomide, a potent TNF-α inhibitor [46], suppressed the sphere formation in the IFIT2 knockdown cells at a non-toxic concentration (Supplementary Figure S3 and Figure 5B). Hence, these results suggest that blocking TNF-α may alleviate the IFIT2 depletion-induced CSC phenotype.

## 4. Discussion

IFIT2 depletion is associated with enhanced EMT, metastasis, and chemoresistance in oral cancer cell lines and poor survival in OSCC patients [31,32,33]. EMT, metastasis, drug resistance, and CSCs are intertwined. Evidently, our present results confirmed that the IFIT2-depleted cells, compared to the sh-control cells, were associated with a higher tumor sphere forming capability, anchorage-independent growth, SP cells, and self-renewal properties, which are congruent with the major hallmarks of CSCs reported in the literature [8,19,47]. The combined assessment of IFIT2 with CSC markers such as CD44 and CD24 may be applied to predict the prognosis of HNC patients. TNF-α may be involved in the IFIT2 depletion-induced CSC-like phenotype.

Tumor sphere-forming cells harbor enhanced metastatic activity, tumorigenicity, drug resistance, and expression of stemness molecules, which reflect their role in the pathogenesis and progression of cancers [48,49]. Therefore, examining the tumor sphere-forming capability is considered a robust technique to identify and isolate CSCs from heterogeneous cancer populations [50]. CSCs have a higher drug efflux capacity, primarily due to the high expression levels and activity of drug transporters such as ABCB1 and ABCG2 [43,44]. SP cells isolated from oral cancer are enriched in stem cell features and chemo/radioresistance compared to non-SP cells [51,52]. In this study, we also observed the presence of SP and an increase in the expression levels of ABCG2, which further exemplifies the stemness property of IFIT2 knockdown cells. Moreover, CSCs enriched in the SP are known to stay in a quiescent state, have intrinsic chemoresistance, evade apoptosis, and acquire chemoresistance upon drug treatment, and our previous study on IFIT2 knockdown cells showed a resistance to multiple drugs, as well as decreased apoptosis upon 5-FU treatment [32].

Emerging studies have started to stratify the CSC population by isolating CD44^high^ and CD24^low^ populations in various cancer types [53,54]. The CD44^high^/CD24^low^ population isolated from OSCC has been reported to overexpress stem cell-related markers, exhibit EMT characteristics, and confer drug resistance via the increased expression of drug transporters, which is consistent with our study, where the IFIT2 knockdown cells present both CD44^high^ and CD24^low^ populations and increased Oct4, Nanog, Nestin, and ABCG2 expression [53,54]. CSC phenotypes are associated with the expression of pluripotency genes and the acquisition of mesenchymal traits, i.e., the loss of E-cadherin and gain of vimentin, which have already been established in IFIT2 knockdown cells [31]. Similarly, increased Oct4 expression in OSCC can modulate tumor-initiating properties via EMT [55]. In breast cancer stem cells, CD24 expression is downregulated by Twist, an EMT molecule, and it is therefore speculated that CD24 downregulation could be an EMT regulator [56]. Moreover, CD24 expression in breast cancer has shown a positive correlation with tumor grading. Our immunohistochemical analysis of CD24 and IFIT2 in OSCC patients showed a strong correlation in their expression pattern, suggesting an important role of CD24 in the CSC-like properties in IFIT2 knockdown cells. Furthermore, emerging evidence also supports the role of IFIT2 depletion in CSCs. The upregulation of IFIT2 can suppress the CSC-like characteristics of radio/chemoresistant breast cancer cells [28].

The IFIT2 knockdown cells showed higher tumorigenicity in an in vivo assay than control cells. These effects were likely due to the lower CD24 expression as the proportion of CD44 expression in the sh-control was comparatively high. CD24 is reported to regulate the gene expression and distribution of tight junction proteins, such as Zonula occludens (ZO)-1, ZO-2, occludin, claudin-7, and par-3, which are associated with the marginal barrier function of epithelial cells and E-cadherin expression in oral epithelial cells derived from human OSCC [57]. The association of E-cadherin and the actin cytoskeleton mediated by catenin and the partitioning defective-3 (par-3)/par-6/atypical PKC polarity complex that is activated by the small GTPase Cdc42 complex mediate the formation of tight junctions initiated by ZO-1 and ZO-2 [58]. Intriguingly, IFIT2-depleted cells showed decreased E-cadherin expression and increased atypical PKC activation, suggesting a possible correlation between CD24 and IFIT2.

Emerging studies have highlighted the role of TNF-α in CSCs. TNF-α treatment could switch the non-CSC (CD44^+^/CD24^+^) population to a CSC-like (CD44^+^/CD24^−^) population by increasing the expression of EMT factors in breast cancer cells [59]. TNF-α stimulates the Snail-driven EMT transition and increases the stemness properties in cholangiocarcinoma and renal cell carcinoma cells [60,61]. Additionally, TNF-α is known to regulate CD44 and its isoform expression in different breast cancer cell lines via a different pathway to promote cell migration [62]. Additionally, TNF-α enhanced the CSC properties in the OSCC via the Notch-Hes1 activation pathway [63]. However, the effect of TNF-α on the CSC phenotype in IFIT2 knockdown requires further investigation. Therefore, the role of enhanced TNF-α expression in regulating an increase in the CSC population/phenotype and facilitating CSC to overcome the immune escape mechanism by elongating cell survival and promoting the malignancy of OSCC requires further validation.

We had tried to overexpress IFIT2 in low IFIT2 expressing cells. Unfortunately, it was difficult to obtain IFIT2-overexpressed cells because the overexpression of IFIT2 triggers apoptosis [64]. Several reports have made similar observations. Altogether, the IFIT2 knockdown cells indeed exhibited an enhanced expression of putative and pluripotent CSC markers and exhibited self-renewal and tumorigenic abilities in the OSCC cells. We are the first to assess the clinical significance of IFIT2 and CSC markers in HNC, including oral cancer patients. Our previous studies have indicated that TNF-α inhibitors can block angiogenesis and metastasis. Here, we further demonstrated that TNF-α may alter the sphere-forming ability. These results suggest that TNF-α may be a therapeutic target in advanced oral cancer patients with low IFIT2 expression.

## 5. Conclusions

The present study demonstrates that IFIT2 depletion promotes CSC-like phenotypes in oral cancer cells. Clinically, IFIT2 and CSC markers may be prognostic factors in HNC patients.

## Figures and Tables

**Table 1:** In vivo tumorigenicity of IFIT2-depleted oral cancer cells.

Cell Number	Subline	Latency (Days)	Tumor Incidence (%)
100,000	sh-control	21	100 (10/10)
	sh-IFIT2-1	21	100 (10/10)
	sh-IFIT2-2	21	100 (10/10)
50,000	sh-control	21	100 (10/10)
	sh-IFIT2-1	21	100 (10/10)
	sh-IFIT2-2	21	100 (10/10)
10,000	sh-control	30	100 (10/10)
	sh-IFIT2-1	28	100 (10/10)
	sh-IFIT2-2	28	100 (10/10)
1000	sh-control	36	100 (10/10)
	sh-IFIT2-1	28	100 (10/10)
	sh-IFIT2-2	32	100 (10/10)
100	sh-control	55	60 (6/10)
	sh-IFIT2-1	48	70 (7/10)
	sh-IFIT2-2	48	80 (8/10)
50	sh-control	68	50 (5/10)
	sh-IFIT2-1	48	70 (7/10)
	sh-IFIT2-2	48	70 (7/10)
25	sh-control	75	16.7 (1/6)
	sh-IFIT2-1	68	66.7 (4/6)
	sh-IFIT2-2	68	50 (3/6)
10	sh-control	114	0 (0/8)
	sh-IFIT2-1	114	25 (2/8)
	sh-IFIT2-2	114	25 (2/8)

## Data Availability

The data that support the findings of this study are available from the corresponding author upon reasonable request.

## Supplementary Materials

The following supporting information can be downloaded at: https://www.mdpi.com/article/10.3390/biomedicines11030896/s1. Table S1: Q-PCR primer; Figure S1: cDNA microarray results of CSC markers in sh-control and stable IFIT2-depleted cells; Figure S2: The protein levels of Nanog and Oct-4 in sh-control and stable IFIT2-depleted cells were determined by Western blotting; Figure S3. Cytotoxicity of pomalidomide in sh-control and stable IFIT2-depleted cells.

## References

[1] Shield K.D., Ferlay J., Jemal A., Sankaranarayanan R., Chaturvedi A.K., Bray F., Soerjomataram I. (2017). The global incidence of lip, oral cavity, and pharyngeal cancers by subsite in 2012. CA Cancer J. Clin..

[2] Bray F., Ferlay J., Soerjomataram I., Siegel R.L., Torre L.A., Jemal A. (2018). Global cancer statistics 2018: GLOBOCAN estimates of incidence and mortality worldwide for 36 cancers in 185 countries. CA Cancer J. Clin..

[3] Shingaki S., Takada M., Sasai K., Bibi R., Kobayashi T., Nomura T., Saito C. (2003). Impact of lymph node metastasis on the pattern of failure and survival in oral carcinomas. Am. J. Surg..

[4] Iyer N.G., Tan D.S., Tan V.K., Wang W., Hwang J., Tan N.C., Sivanandan R., Tan H.K., Lim W.T., Ang M.K. (2015). Randomized trial comparing surgery and adjuvant radiotherapy versus concurrent chemoradiotherapy in patients with advanced, nonmetastatic squamous cell carcinoma of the head and neck: 10-year update and subset analysis. Cancer.

[5] Jerjes W., Upile T., Petrie A., Riskalla A., Hamdoon Z., Vourvachis M., Karavidas K., Jay A., Sandison A., Thomas G.J. (2010). Clinicopathological parameters, recurrence, locoregional and distant metastasis in 115 T1-T2 oral squamous cell carcinoma patients. Head Neck Oncol..

[6] Baillie R., Tan S.T., Itinteang T. (2017). Cancer Stem Cells in Oral Cavity Squamous Cell Carcinoma: A Review. Front. Oncol..

[7] Reya T., Morrison S.J., Clarke M.F., Weissman I.L. (2001). Stem cells, cancer, and cancer stem cells. Nature.

[8] Chiou S.H., Yu C.C., Huang C.Y., Lin S.C., Liu C.J., Tsai T.H., Chou S.H., Chien C.S., Ku H.H., Lo J.F. (2008). Positive correlations of Oct-4 and Nanog in oral cancer stem-like cells and high-grade oral squamous cell carcinoma. Clin. Cancer Res..

[9] Mani S.A., Guo W., Liao M.J., Eaton E.N., Ayyanan A., Zhou A.Y., Brooks M., Reinhard F., Zhang C.C., Shipitsin M. (2008). The epithelial-mesenchymal transition generates cells with properties of stem cells. Cell.

[10] Gupta P.B., Pastushenko I., Skibinski A., Blanpain C., Kuperwasser C. (2019). Phenotypic Plasticity: Driver of Cancer Initiation, Progression, and Therapy Resistance. Cell Stem Cell.

[11] Visvader J.E., Lindeman G.J. (2008). Cancer stem cells in solid tumours: Accumulating evidence and unresolved questions. Nat. Rev. Cancer.

[12] Eramo A., Lotti F., Sette G., Pilozzi E., Biffoni M., Di Virgilio A., Conticello C., Ruco L., Peschle C., De Maria R. (2008). Identification and expansion of the tumorigenic lung cancer stem cell population. Cell Death Differ..

[13] Maitland N.J., Collins A.T. (2008). Prostate cancer stem cells: A new target for therapy. J. Clin. Oncol..

[14] Li C., Heidt D.G., Dalerba P., Burant C.F., Zhang L., Adsay V., Wicha M., Clarke M.F., Simeone D.M. (2007). Identification of pancreatic cancer stem cells. Cancer Res..

[15] Han J., Fujisawa T., Husain S.R., Puri R.K. (2014). Identification and characterization of cancer stem cells in human head and neck squamous cell carcinoma. BMC Cancer.

[16] Pohl A., Lurje G., Kahn M., Lenz H.J. (2008). Stem cells in colon cancer. Clin. Color. Cancer.

[17] Batlle E., Sancho E., Franci C., Dominguez D., Monfar M., Baulida J., Garcia De Herreros A. (2000). The transcription factor snail is a repressor of E-cadherin gene expression in epithelial tumour cells. Nat. Cell Biol..

[18] Cano A., Perez-Moreno M.A., Rodrigo I., Locascio A., Blanco M.J., del Barrio M.G., Portillo F., Nieto M.A. (2000). The transcription factor snail controls epithelial-mesenchymal transitions by repressing E-cadherin expression. Nat. Cell Biol..

[19] Baniebrahimi G., Mir F., Khanmohammadi R. (2020). Cancer stem cells and oral cancer: Insights into molecular mechanisms and therapeutic approaches. Cancer Cell Int..

[20] Fensterl V., Sen G.C. (2011). The ISG56/IFIT1 gene family. J. Interferon Cytokine Res..

[21] Diamond M.S., Farzan M. (2013). The broad-spectrum antiviral functions of IFIT and IFITM proteins. Nat. Rev. Immunol..

[22] Pidugu V.K., Pidugu H.B., Wu M.M., Liu C.J., Lee T.C. (2019). Emerging Functions of Human IFIT Proteins in Cancer. Front. Mol. Biosci..

[23] Zhou X., Michal J.J., Zhang L., Ding B., Lunney J.K., Liu B., Jiang Z. (2013). Interferon induced IFIT family genes in host antiviral defense. Int. J. Biol. Sci..

[24] Terenzi F., White C., Pal S., Williams B.R., Sen G.C. (2007). Tissue-specific and inducer-specific differential induction of ISG56 and ISG54 in mice. J. Virol..

[25] Pidugu V.K., Wu M.M., Yen A.H., Pidugu H.B., Chang K.W., Liu C.J., Lee T.C. (2019). IFIT1 and IFIT3 promote oral squamous cell carcinoma metastasis and contribute to the anti-tumor effect of gefitinib via enhancing p-EGFR recycling. Oncogene.

[26] Lo U.G., Bao J., Cen J., Yeh H.C., Luo J., Tan W., Hsieh J.T. (2019). Interferon-induced IFIT5 promotes epithelial-to-mesenchymal transition leading to renal cancer invasion. Am. J. Clin. Exp. Urol..

[27] Zhao Y., Altendorf-Hofmann A., Pozios I., Camaj P., Daberitz T., Wang X., Niess H., Seeliger H., Popp F., Betzler C. (2017). Elevated interferon-induced protein with tetratricopeptide repeats 3 (IFIT3) is a poor prognostic marker in pancreatic ductal adenocarcinoma. J. Cancer Res. Clin. Oncol..

[28] Koh S.Y., Moon J.Y., Unno T., Cho S.K. (2019). Baicalein Suppresses Stem Cell-Like Characteristics in Radio- and Chemoresistant MDA-MB-231 Human Breast Cancer Cells through Up-Regulation of IFIT2. Nutrients.

[29] Chen L., Zhai W., Zheng X., Xie Q., Zhou Q., Tao M., Zhu Y., Wu C., Jiang J. (2018). Decreased IFIT2 Expression Promotes Gastric Cancer Progression and Predicts Poor Prognosis of the Patients. Cell. Physiol. Biochem..

[30] Su W., Xiao W., Chen L., Zhou Q., Zheng X., Ju J., Jiang J., Wang Z. (2019). Decreased IFIT2 Expression In Human Non-Small-Cell Lung Cancer Tissues Is Associated With Cancer Progression And Poor Survival Of The Patients. OncoTargets Ther..

[31] Lai K.C., Liu C.J., Chang K.W., Lee T.C. (2013). Depleting IFIT2 mediates atypical PKC signaling to enhance the migration and metastatic activity of oral squamous cell carcinoma cells. Oncogene.

[32] Regmi P., Lai K.C., Liu C.J., Lee T.C. (2020). SAHA Overcomes 5-FU Resistance in IFIT2-Depleted Oral Squamous Cell Carcinoma Cells. Cancers.

[33] Lai K.C., Chang K.W., Liu C.J., Kao S.Y., Lee T.C. (2008). IFN-induced protein with tetratricopeptide repeats 2 inhibits migration activity and increases survival of oral squamous cell carcinoma. Mol. Cancer Res..

[34] Zhang Z., Li N., Liu S., Jiang M., Wan J., Zhang Y., Wan L., Xie C., Le A. (2020). Overexpression of IFIT2 inhibits the proliferation of chronic myeloid leukemia cells by regulating the BCRABL/AKT/mTOR pathway. Int. J. Mol. Med..

[35] Chen L., Liu S., Xu F., Kong Y., Wan L., Zhang Y., Zhang Z. (2017). Inhibition of Proteasome Activity Induces Aggregation of IFIT2 in the Centrosome and Enhances IFIT2-Induced Cell Apoptosis. Int. J. Biol. Sci..

[36] Feng X., Wang Y., Ma Z., Yang R., Liang S., Zhang M., Song S., Li S., Liu G., Fan D. (2014). MicroRNA-645, up-regulated in human adencarcinoma of gastric esophageal junction, inhibits apoptosis by targeting tumor suppressor IFIT2. BMC Cancer.

[37] Ohsugi T., Yamaguchi K., Zhu C., Ikenoue T., Furukawa Y. (2017). Decreased expression of interferon-induced protein 2 (IFIT2) by Wnt/beta-catenin signaling confers anti-apoptotic properties to colorectal cancer cells. Oncotarget.

[38] Lai K.C., Liu C.J., Lin T.J., Mar A.C., Wang H.H., Chen C.W., Hong Z.X., Lee T.C. (2016). Blocking TNF-alpha inhibits angiogenesis and growth of IFIT2-depleted metastatic oral squamous cell carcinoma cells. Cancer Lett..

[39] Du F., Liu H., Lu Y., Zhao X., Fan D. (2017). Epithelial-to-Mesenchymal Transition: Liaison between Cancer Metastasis and Drug Resistance. Crit. Rev. Oncog..

[40] Niess H., Camaj P., Renner A., Ischenko I., Zhao Y., Krebs S., Mysliwietz J., Jackel C., Nelson P.J., Blum H. (2015). Side population cells of pancreatic cancer show characteristics of cancer stem cells responsible for resistance and metastasis. Target Oncol..

[41] Hamburger A.W., Salmon S.E. (1977). Primary bioassay of human tumor stem cells. Science.

[42] Song J., Chang I., Chen Z., Kang M., Wang C.Y. (2010). Characterization of side populations in HNSCC: Highly invasive, chemoresistant and abnormal Wnt signaling. PLoS ONE.

[43] Greve B., Kelsch R., Spaniol K., Eich H.T., Gotte M. (2012). Flow cytometry in cancer stem cell analysis and separation. Cytometry A.

[44] Hirschmann-Jax C., Foster A.E., Wulf G.G., Nuchtern J.G., Jax T.W., Gobel U., Goodell M.A., Brenner M.K. (2004). A distinct “side population” of cells with high drug efflux capacity in human tumor cells. Proc. Natl. Acad. Sci. USA.

[45] Nagy Á., Munkácsy G., Győrffy B. (2021). Pancancer survival analysis of cancer hallmark genes. Sci. Rep..

[46] Wang F., Liu W., Jiang Q., Gong M., Chen R., Wu H., Han R., Chen Y., Han D. (2019). Lipopolysaccharide-induced testicular dysfunction and epididymitis in mice: A critical role of tumor necrosis factor alphadagger. Biol. Reprod..

[47] Shrivastava S., Steele R., Sowadski M., Crawford S.E., Varvares M., Ray R.B. (2015). Identification of molecular signature of head and neck cancer stem-like cells. Sci. Rep..

[48] Reynolds D.S., Tevis K.M., Blessing W.A., Colson Y.L., Zaman M.H., Grinstaff M.W. (2017). Breast Cancer Spheroids Reveal a Differential Cancer Stem Cell Response to Chemotherapeutic Treatment. Sci. Rep..

[49] Morata-Tarifa C., Jimenez G., Garcia M.A., Entrena J.M., Grinan-Lison C., Aguilera M., Picon-Ruiz M., Marchal J.A. (2016). Low adherent cancer cell subpopulations are enriched in tumorigenic and metastatic epithelial-to-mesenchymal transition-induced cancer stem-like cells. Sci. Rep..

[50] Cao L., Zhou Y., Zhai B., Liao J., Xu W., Zhang R., Li J., Zhang Y., Chen L., Qian H. (2011). Sphere-forming cell subpopulations with cancer stem cell properties in human hepatoma cell lines. BMC Gastroenterol..

[51] Zhang P., Zhang Y., Mao L., Zhang Z., Chen W. (2009). Side population in oral squamous cell carcinoma possesses tumor stem cell phenotypes. Cancer Lett..

[52] Yanamoto S., Kawasaki G., Yamada S., Yoshitomi I., Kawano T., Yonezawa H., Rokutanda S., Naruse T., Umeda M. (2011). Isolation and characterization of cancer stem-like side population cells in human oral cancer cells. Oral Oncol..

[53] Todoroki K., Ogasawara S., Akiba J., Nakayama M., Naito Y., Seki N., Kusukawa J., Yano H. (2016). CD44v^3+^/CD24^−^ cells possess cancer stem cell-like properties in human oral squamous cell carcinoma. Int. J. Oncol..

[54] Ghuwalewala S., Ghatak D., Das P., Dey S., Sarkar S., Alam N., Panda C.K., Roychoudhury S. (2016). CD44(high)CD24(low) molecular signature determines the Cancer Stem Cell and EMT phenotype in Oral Squamous Cell Carcinoma. Stem Cell Res.

[55] Tsai L.L., Hu F.W., Lee S.S., Yu C.H., Yu C.C., Chang Y.C. (2014). Oct4 mediates tumor initiating properties in oral squamous cell carcinomas through the regulation of epithelial-mesenchymal transition. PLoS ONE.

[56] Vesuna F., Lisok A., Kimble B., Raman V. (2009). Twist modulates breast cancer stem cells by transcriptional regulation of CD24 expression. Neoplasia.

[57] Ye P., Nadkarni M.A., Hunter N. (2006). Regulation of E-cadherin and TGF-beta3 expression by CD24 in cultured oral epithelial cells. Biochem. Biophys. Res. Commun..

[58] Schneeberger E.E., Lynch R.D. (2004). The tight junction: A multifunctional complex. Am. J. Physiol. Cell Physiol..

[59] Bhat-Nakshatri P., Appaiah H., Ballas C., Pick-Franke P., Goulet R., Badve S., Srour E.F., Nakshatri H. (2010). SLUG/SNAI2 and tumor necrosis factor generate breast cells with CD44^+^/CD24^−^ phenotype. BMC Cancer.

[60] Techasen A., Namwat N., Loilome W., Bungkanjana P., Khuntikeo N., Puapairoj A., Jearanaikoon P., Saya H., Yongvanit P. (2012). Tumor necrosis factor-alpha (TNF-alpha) stimulates the epithelial-mesenchymal transition regulator Snail in cholangiocarcinoma. Med. Oncol..

[61] Zhang L., Jiao M., Wu K., Li L., Zhu G., Wang X., He D., Wu D. (2014). TNF-alpha induced epithelial mesenchymal transition increases stemness properties in renal cell carcinoma cells. Int. J. Clin. Exp. Med..

[62] Li J., Zha X.M., Wang R., Li X.D., Xu B., Xu Y.J., Yin Y.M. (2012). Regulation of CD44 expression by tumor necrosis factor-alpha and its potential role in breast cancer cell migration. Biomed. Pharmacother..

[63] Lee S.H., Hong H.S., Liu Z.X., Kim R.H., Kang M.K., Park N.H., Shin K.H. (2012). TNFα enhances cancer stem cell-like phenotype via Notch-Hes1 activation in oral squamous cell carcinoma cells. Biochem. Biophys. Res. Commun..

[64] Reich N.C. (2013). A death-promoting role for ISG54/IFIT2. J. Interferon Cytokine Res..

