# Supplementary material for: IFIT2 Depletion Promotes Cancer Stem Cell-like Phenotypes in Oral Cancer
Source: Biomedicines. 2023 Mar 14;11(3):896. doi: 10.3390/biomedicines11030896 (PMC10045464; doi:10.3390/biomedicines11030896)
Supplement: Supplementary file 1 [file biomedicines-11-00896-s001.zip › SUPLEMENTARY RESULTS/FIGURE S1.pdf]

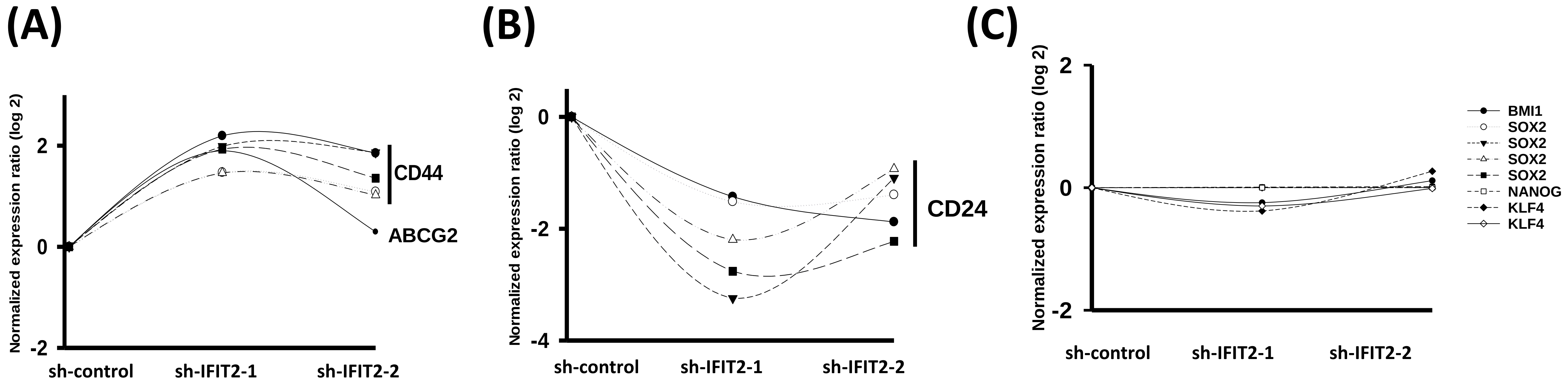


Figure S1. cDNA microarray results of CSC markers in sh-control, sh-IFIT2-1, and sh-IFIT2-2 cells. (A) Enhanced CD44 and ABCG2 and (B) Reduced CD24 were observed in sh-IFIT2-1 and sh-IFIT2-2 cells compared to sh-control cells. (C) Other CSC markers were not significantly different between IFIT2 knockdown and control cells. Each line represents an array probe.
