# Supplementary material for: IFIT2 Depletion Promotes Cancer Stem Cell-like Phenotypes in Oral Cancer
Source: Biomedicines. 2023 Mar 14;11(3):896. doi: 10.3390/biomedicines11030896 (PMC10045464; doi:10.3390/biomedicines11030896)
Supplement: Supplementary file 1 [file biomedicines-11-00896-s001.zip › SUPLEMENTARY RESULTS/FIGURE S2.pdf]

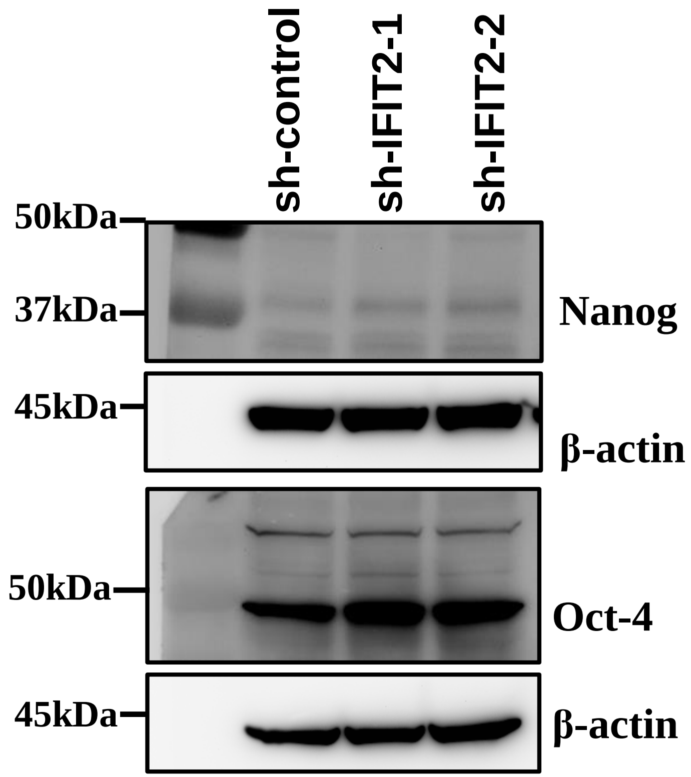

Figure S2. The protein levels of Nanog and Oct-4 in sh-control, sh-IFIT2-1 and sh-IFIT2-2 cells were determined by Western blotting. β-actin was used as an internal control Western blotting.
