# Supplementary material for: IFIT2 Depletion Promotes Cancer Stem Cell-like Phenotypes in Oral Cancer
Source: Biomedicines. 2023 Mar 14;11(3):896. doi: 10.3390/biomedicines11030896 (PMC10045464; doi:10.3390/biomedicines11030896)
Supplement: Supplementary file 1 [file biomedicines-11-00896-s001.zip › SUPLEMENTARY RESULTS/FIGURE S3.pdf]

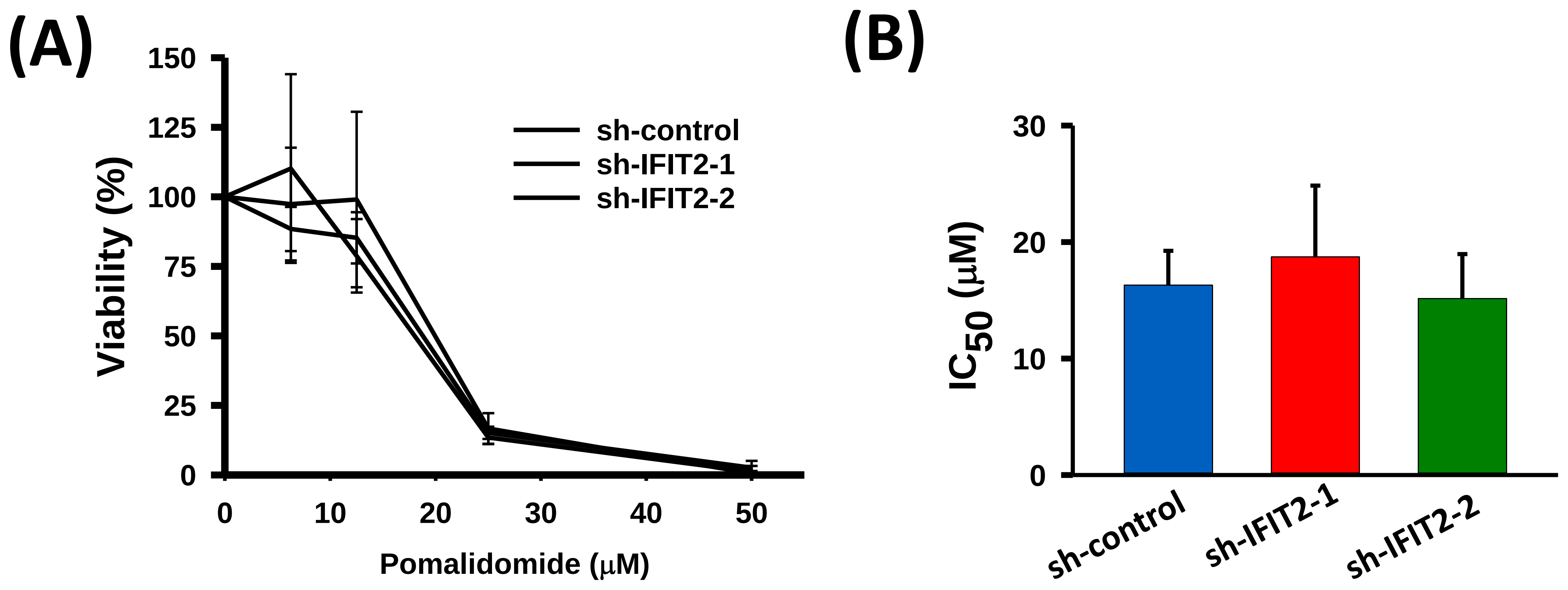

Figure S3. Cytotoxicity of pomalidomide in sh-control, sh-IFIT2-1, and sh-IFIT2-2 cells. (A) The cytotoxicity effect of pomalidomide was assessed by seeding 3000 cells into each well of a 96-well plate, followed by incubation overnight and treatment with various concentrations of pomalidomide for 72 h. The cell growth was determined by PrestoBlue (Invitrogen, Carlsbad, CA, USA) incubation; (B)The IC_{50} value of pomalidomide was calculated using Compusyn software (version 1.0.1; CompuSyn, Inc., Paramus, NJ, USA). There was no significant difference in IC_{50} among these three cells. The quantitative data are from three independent experiments.
