# Supplementary material for: IFIT2 Depletion Promotes Cancer Stem Cell-like Phenotypes in Oral Cancer
Source: Biomedicines. 2023 Mar 14;11(3):896. doi: 10.3390/biomedicines11030896 (PMC10045464; doi:10.3390/biomedicines11030896)
Supplement: Supplementary file 1 [file biomedicines-11-00896-s001.zip › SUPLEMENTARY RESULTS/Table S1_KCL.pdf]

Table S1. Primer sequences of CSC markers for quantitative real-time PCR analysis.

**Gene**	**Forward primer**	**Reverse primer**
OCT3/4	5-GAAGGTATTCAGCCAAACGA-3'	5-AAATTCTCCAGGTTGCCTCT-3'
NANOG	5-ACCAGACCTGGAACAAGTTCA-3'	5-ATGCAGGACTGCAGAGATTC-3'
NESTIN	5-CATGGAACCTGGAGAATTTG-3'	5-AGCCAGTTCTTGGTCCTTCT-3'
ABCG2	5-GTTTTTCCCTGACATCGTGGA-3'	5-CGAGCCTCTTGGTATAGGCG-3'
CD24	5-ACAGCCAGTCTCTTCGTGGT-3’	5-CCTGTTTTTCCTTGCCACAT-3’
CD44	5-CGGACACCATGGACAAGTTT-3’	5-CCGTCCGAGAGATGCTGTAG-3’
GAPDH	5-GGAGTCCCTGCCACACTCA-3'	5-GCCCCTCCCCTCTTCAAG-3'
